# Association of *MAOA* genetic variants and resilience with psychosocial stress: A longitudinal study of Syrian refugees

**DOI:** 10.1371/journal.pone.0219385

**Published:** 2019-07-17

**Authors:** Christopher J. Clukay, Rana Dajani, Kristin Hadfield, Jacklyn Quinlan, Catherine Panter-Brick, Connie J. Mulligan

**Affiliations:** 1 Department of Anthropology, University of Florida, Gainesville, FL, United States of America; 2 Genetics Institute, University of Florida, Gainesville, FL, United States of America; 3 Department of Biology and Biotechnology, Hashemite University, Zarqa, Jordan; 4 School of Biological and Chemical Sciences, Queen Mary, University of London, London, England; 5 Department of Anthropology, Yale University, New Haven, CT, United States of America; Stellenbosch University, SOUTH AFRICA

## Abstract

Early childhood trauma can have profound and lifelong effects on adult mental health and psychosocial wellbeing. Nevertheless, responses to trauma are highly variable. Genetic variants may help explain variation in responses to trauma by identifying alleles that associate with changes in mental health measures. Protective factors, such as resilience, likely also play an important role in responses to trauma. The effects of genetic variants, in combination with protective factors, on psychosocial health are not well understood, particularly in non-Western contexts. In this study, we test the relative influence of genetic variants of monoamine oxidase A (*MAOA*, a gene proposed to influence the impact of childhood trauma on adult violence and antisocial behavior), levels of resilience, and exposure to traumatic events on psychosocial stress and mental health trajectories over time. We use data from a cohort of 12-18-year-old Syrian refugees who were forcibly displaced to neighboring Jordan (*n* = 399). DNA samples and survey data on trauma exposure, resilience (CYRM-12), and psychosocial stress were collected at three time points: baseline, ~13 weeks, and ~48 weeks. Using multilevel models, we identified an association of *MAOA* variant, in males only, with symptom scores of psychosocial stress on the Perceived Stress Scale (PSS) over time (*p* = 8.1 x 10^−4^). We also found that resilience is strongly associated with PSS (*p* = 7.9 x 10^−9^), underscoring the importance of protective factors in influencing levels of psychosocial stress. Furthermore, there was an additive effect wherein the sharpest reductions in perceived psychosocial stress are seen in low-activity *MAOA* males with low trauma exposure or high resilience levels. Our results highlight the value of studies that integrate genetic and psychosocial factors to better understand complex phenotypes, such as responses to trauma in contexts of high trauma exposure.

## Introduction

Childhood traumatic experiences are associated with poor health outcomes that can persist throughout life. In particular, children and adolescents who experience physical and emotional abuse are at increased risk for psychiatric diagnoses, especially mood, anxiety, and substance abuse disorders, as well as suicidality, violent and criminal behavior, poor physical health and increased risk for infectious disease later in life [1–7]. However, there is great variation in how children experience and recover from early trauma [8]. There is a paucity of longitudinal research conducted outside Western contexts on this issue, especially with war-affected children and adolescents. Research on refugee populations is of great importance because refugees experience a high burden of trauma, loss, and stress [9]. Understanding how war-related adversity and trauma impacts health and development is particularly critical in children and adolescents since they account for 52% of refugees worldwide [10]. Many refugees are exposed to substantial trauma during war, as well as ongoing stressors and social marginalization during and after resettlement. Nevertheless, many refugees show notable resilience in psychosocial, developmental, and health trajectories [11, 12]. Now in its seventh year, the Syrian crisis has led more than 5.68 million people to leave Syria, while a further 6.5 million Syrians are internally displaced, two-fifths of whom are under 18 years of age [13, 14]. This is the largest refugee crisis since the Second World War, the consequences of which have led scholars and humanitarian agencies to call for renewed attention to address health and developmental issues for a potentially ‘lost generation’ coping with exposures to ‘toxic stress.’ [9].

The factors that determine how children experience and recover from trauma are not well understood, but likely include both genetic and psychosocial factors [8]. The monoamine oxidase A gene (*MAOA*) has been widely studied for its role in influencing the impact of childhood trauma on adult aggressive and antisocial behavior. *MAOA* is located on the X chromosome and the encoded MAOA enzyme catalyzes the degradation of brain neurotransmitters serotonin, dopamine and norepinephrine, thus playing a key role in regulating behavior. A functional repeat polymorphism located approximately 1.2 kb upstream of the *MAOA* coding sequence was first identified by Sabol et al. [15], who used luciferase reporter gene fusions and transfection experiments to show that alleles with different numbers of repeat sequences were transcribed with different efficiencies in human neuroblastoma and human placental choriocarcinoma cell lines. MAOA deficiency was first studied in humans in five affected males who exhibited borderline mental deficiency as well as impulsive aggression, criminal violence, and hypersexual behavior [16]. In the early 2000’s, Caspi and Moffitt’s [17] landmark Dunedin Longitudinal Cohort study investigated *MAOA* genetic variants to better understand why some abused children developed antisocial behavior as adults and others did not. They determined that childhood trauma was more likely to lead to antisocial and violent behavior in males who carried a *MAOA* variant with low transcriptional activity (*MAOA-L*) [17].

Over the past decade, additional studies have identified associations between *MAOA* variants and a wide range of aggressive and antisocial behaviors including adolescent antisocial behavior [18], reduced social cooperation [19], physical aggression [20], criminal violence [21], recidivism in male violent offenders [22], and brain morphology in violent offenders with psychopathic traits [23]. Not all associations have been confirmed, however, and the first genome-wide association study on adult antisocial behavior did not find significant associations with any genetic polymorphisms, leading the authors to speculate that the relevant genes may have effect sizes which are so small that they are undetectable with their sample size [24]. In general, *MAOA-L* is the allele most often associated with increased aggressive or antisocial behaviors and the strongest associations are typically found in males [25, 26].

Interestingly, some studies have found opposite effects in females, i.e. association of the high-activity *MAOA* (*MAOA-H*) variant with increased risk of antisocial behavior and aggression [25, 27]. A study of neural activity found that activity in the amygdala and hippocampus increased with childhood stress in *MAOA-L* males and decreased in *MAOA-H* males, with the opposite pattern seen in females [27]. Another brain imaging study found increased connectivity between the amygdala and ventromedial prefrontal cortex that indicated dysregulated amygdala activation in *MAOA-L* males; this effect was not seen in females [28]. Variable levels of testosterone and the specific interaction of *MAOA-L* with high levels of testosterone [29] likely explain some of the differing effects of *MAOA* between males and females, and possibly within males as well.

The timing of trauma exposure is critical since brain structures are still developing during childhood and adolescence; disruption of different brain structures will likely impact different psychosocial outcomes [30, 31]. The critical developmental periods, and sensitive windows, for three key regions of the brain (hippocampus, amygdala, prefrontal cortex) all overlap in early and middle childhood [32, 33]. Children across a range of ages have been studied in order to investigate critical windows of trauma exposure. For instance, family adversity experienced in the first three years of life has been found to associate with increased hyperactivity at 4–7 years of age in *MAOA-L* boys and girls from the U.K. Avon Longitudinal Study of Parents and Children, UK [34]. Fewer studies have focused on adolescents, but Lee [18] found that the effect of deviant peer affiliation on antisocial behavior in participants from the National Longitudinal Study of Adolescent Health study was significantly stronger in *MAOA-H* carriers than *MAOA-L* carriers. In sum, different studies identify both *MAOA-L* and *MAOA-H* variants as being risk alleles, which may reflect ongoing brain development and exposure to trauma at different ages.

In addition to the effect of *MAOA* on aggressive and antisocial behaviors, other behaviors have been tested for association with *MAOA* in order to provide a more comprehensive perspective on MAOA functioning and to better understand the behaviors impacted by *MAOA*. For instance, *MAOA-L* variants have been found at slightly higher frequencies in alcoholics (both male and female) with antisocial personality disorder compared to alcoholics with anxiety and depression, suggesting that *MAOA* variation may contribute to over- vs under-reactive behaviors in the development of alcoholism [26]. *MAOA* likely also plays a role in the risk of developing stress-related behaviors like obsessive-compulsive disorder [35] as well as addictive behaviors like cigarette smoking [36]. In a study on assessment of risk in a laboratory setting, *MAOA-H* individuals showed a preference for longshot risks and were less likely to purchase insurance, suggesting that *MAOA* variation may play a role in real-life decision-making and risk-assessment [37]. Negative associations can also be informative, such as the study that found *MAOA* was not one of the nine candidate genes associated with response time on an attention task, suggesting that *MAOA* does not play a role in attentional deficits [38]. Overall, our understanding of the effect of *MAOA* on complex behaviors is incomplete, but the impact of *MAOA* is likely to be relatively small, and possibly difficult to detect [39].

Virtually no studies have investigated the influence of *MAOA* and protective factors on psychosocial and mental health outcomes. We found a single published study of *MAOA* and a protective factor: high perceived parental care was found to mitigate the effect of childhood stress in females (males were not studied) [40]. Specifically, a three-way interaction between perceived parental care, early life family stressors, and *MAOA* variant predicted impulsivity/aggression scores. The lack of attention to protective factors is puzzling given the strong effect of protective factors on mental and physical health [41–46] and the opportunity for intervention [47, 48] with potential for intergenerational effects [49, 50]. Furthermore, from an evolutionary perspective, it is unlikely to find common genetic variants whose only effect is to increase the risk of maladaptive behaviors under adverse conditions. Pluess and Belsky have proposed that genetic variants like those at *MAOA* do not function solely as risk factors, but rather as environmental sensitivity variants whose carriers are more sensitive, or responsive, to both positive and negative exposures [51–53]. In their brain imaging study, Buckholtz et al. [54] proposed that *MAOA-L* variants may create a less stable developmental framework, e.g. amygdala dysregulation and a compensatory response of increased coupling between the amygdala and the ventromedial prefrontal cortex, that renders *MAOA-L* males more sensitive to environmental factors relative to *MAOA-H* males.

In this study, we tested for association of *MAOA* variants with different measures of psychosocial stress and mental health in a population of Syrian refugee youth who have experienced high levels of war-related trauma. We also investigated the protective aspects of a contextually-specific measure of resilience that measures individual and collective strengths, resources, and capabilities [55]. The goal in studying a range of measures was to identify outcomes other than aggressive and antisocial behavior that associate with *MAOA*. We predicted there would be an association of *MAOA* genetic variants, either directly or through an interaction with trauma exposure or resilience, with psychosocial outcomes. Specifically, we hypothesized that *MAOA-L* individuals would show increased change in the measured outcomes relative to *MAOA-H* carriers and that this effect would be limited to males, based on findings of dysregulated amygdala activation and proposed increased environmental sensitivity in *MAOA-L* males [28, 56].

## Materials and methods

### Study design

Our study population was a cohort of 399 12-18-year-old Syrian refugees in Jordan. Over 671,000 Syrians have taken refuge in Jordan [14]. Participating adolescents were sampled from four sites in northern Jordan (Irbid, Jarash, Mafraq, Zarqa), urban centers which have been heavily affected by the Syrian crisis. Youth in our study were participating in an eight-week stress attunement intervention (*Advancing Adolescents*; Arabic: *Nubader*), developed by Mercy Corps for war-affected adolescents in Jordan, Lebanon, Iraq, Syria and Turkey. Buccal samples and data on trauma exposure, resilience, stress, and mental health were collected at three time points: pre-intervention baseline (T1), after ~13 weeks (T2), and after ~48 weeks (T3). Refugees at these four study sites were from disadvantaged families and most were displaced from the cities of Damascus, Daraa, Homs, and surrounding towns in Syria. In order to control for possible population structure created by study sites having different proportions of refugees from different areas of Syria, study site was included as a covariate in all analyses. Participants were sampled in two waves: in the first wave (collected March-June 2015, *n* = 103), participants were quasi-randomized into treatment groups based on their availability to participate in the intervention immediately, and in the second wave (collected September 2015-February 2016, *n* = 296), participants were randomly allocated to treatment or wait-list. There were no significant differences in our independent variables (*MAOA* variant and trauma exposure; data on resilience were collected in the second wave only), enabling us to combine the two waves in order to increase sample size. In this paper, we focus on the 399 adolescents (221 males and 178 females) for whom biological samples were collected. The study received approval from the Prime Minister’s Office of Jordan as well as ethical approval from Yale University (IRB ID 1502015359). Informed consent was obtained in Arabic from all participating adolescents and their parents. No participant who was asked to contribute a buccal sample refused to do so. See previous publications [57–59] for more details on the study method. All data analyzed in this study are presented in S1 and S2 Tables. All data are also available on Mendeley (https://data.mendeley.com/datasets/) doi:10.17632/xhs8tn7nkz.1).

### Study population

Individuals with buccal samples and data on trauma exposure, resilience, and the outcome measures were chosen for study (T1 = 399) (Table 1). Some study participants were lost to follow-up at the T2 and T3 time points, resulting in a sample of 263 participants at T2 and 157 participants at T3. Because multilevel models are robust to partial data [60], individuals with missing time points were included in all analyses; thus, each data point contributes to the model.

**Table 1:** Sample characteristics of Syrian refugee participants.

	Male	Female
**Sociodemographics**		
# Participants at T1	221	178
# Participants at T2	136	127
# Participants at T3	78	79
Age, mean (SD) years	14.2 (1.81)	14.5 (1.88)
**Genetic Data**		
*MAOA-H*, # Homozygotes or Hemizygotes	125	50
*MAOA-H/L*, # Heterozygotes	NA	94
*MAOA-L*, # Homozygotes or Hemizygotes	96	34
**Trauma exposure and Resilience Measures**		
Lifetime Exposure to Traumatic Events* (SD)	6.76 (3.25)	5.81 (3.21)
Resilience, CYRM12 (SD)	49.8 (6.77)	49.1 (7.00)
**Stress and Mental Health Measures**		
Perceived Stress Scale, PSS* (SD)	28.1 (5.37)	29.4 (6.38)
Human Distress Scale, HD (SD)	39.7 (19.3)	44.5 (22.7)
Human Insecurity Scale, HI* (SD)	66.3 (20.0)	70.0 (19.2)
Arab Youth Mental Health Scale, AYMH* (SD)	34.6 (7.72)	37.2 (9.25)
Strengths & Difficulties Questionnaire, SDQ (SD)	14.9 (5.68)	16.3 (6.23)
Children’s Revised Impact of Events Scale, CRIES-8 (SD)	19.6 (10.6)	19.1 (11.7)

Values are reported as mean with standard deviation in parentheses and are from baseline. An asterisk (*) indicates significant differences between males and females at *p* < 0.05. For *MAOA*, repeat lengths 3.5, 4, and 5 were classified as high-activity variants and repeat lengths 2 and 3 were classified as low-activity variants. Since males have only one copy of the X-linked *MAOA* gene, there were no male heterozygotes. Resilience measures were collected for a subset of 163 males and 127 females.

### Psychosocial measures

Lifetime trauma exposure was assessed with the Traumatic Events Checklist, adapted for use with adolescents in conflict regions [61]. Resilience was measured using the Child and Youth Resilience Measure (CYRM) that was specifically developed to assess resilience in youth living in adversity [55]. This measure of resilience is situated within a socioecological framework [55, 62] and assesses protective factors at individual, relational, and contextual levels, rather than treating resilience as an internal trait or using better-than-expected functioning as a proxy for resilience. Since the resilience measure was developed during this study, it was only collected in the second wave of survey with 163 males and 127 females.

We tested six psychosocial and mental health measures. The Perceived Stress Scale (PSS, 14 items) is a generalized measure of psychosocial stress used globally that has been validated in Jordan [63, 64]. Human Distress (HD, 12 items) is a measure of distress and Human Insecurity (HI, 10 items) is a measure of insecurity and captures feelings of fear in conflict-affected areas. Both HD and HI were developed for use in conflict settings in the Middle East and were validated in the West Bank [65]. The Arab Youth Mental Health scale (AYMH, 21 items) is used to screen for depression and anxiety in Arab youth [66]. The Strength and Difficulties Questionnaire (SDQ, 25 items) assesses behavioral and emotional mental health difficulties [67–69]. Finally, the Children’s Revised Impact of Event Scale (CRIES-8, 8 items) measures symptoms of posttraumatic stress [70, 71].

### Buccal sample collection and DNA extraction

Buccal samples were collected using Transport Swabs (APCO Laboratory Consumable Plastic, Jordan) or DNA Buccal Swabs (Isohelix, United Kingdom). Participants rinsed their mouths with water and then brushed both sides of their mouth with the collection swab for up to 30 seconds. DNA was extracted from the buccal swabs using either the Qiagen DNA Investigator Kit (Qiagen, USA) or Xtreme DNA Isolation Kit (Isohelix, United Kingdom). DNA extractions were performed according to manufacturer’s recommendations with the exception that the AW2 wash was performed twice for swabs extracted using the Qiagen kit.

### *MAOA* assay

Samples were PCR amplified based on a published protocol [34]. All samples used recommended reagent concentrations including 1uL of extracted DNA, Platinum Hot Start PCR Master Mix and Platinum GC Enhancer (Invitrogen, USA), and the following primer sequences: Forward 5’-CCC-AGG-CTG-CTC-CAG-AAA-C-3’ and Reverse 5’-ACT-CAG-AAC-GGA-TGC-TCC-ATT-CG-3’. The PCR profile was as follows: 96°C for 10 min followed by 40 cycles of 94°C for 15 sec, 55°C for 15 sec, and 72°C for 30 sec and a final 3 min step at 72°C. Amplification reactions were electrophoresed on 2% agarose gels using Agarose SFR (VWR Life Science, USA) for 3 hours at 80V. Genotype completion rate was 97%. Genotyping discrepancy was determined by replicating approximately 1/3 of the samples (n = 153); only one sample showed a discrepancy and was typed a third time to resolve the discrepancy.

### Statistical analyses

Each *MAOA* variant was classified according to transcriptional activity: repeat lengths of 3.5, 4, and 5 were classified as high-activity variants while repeat lengths of 2 and 3 were classified as low-activity variants. Since *MAOA* occurs on the X chromosome, males are hemizygous for *MAOA*, i.e. they carry only one *MAOA* variant. In statistical analyses, high-activity male hemizygotes and high-activity female homozygotes were given a score of 1, female heterozygotes with one high-activity and one low-activity variant were given a score of 0.5, and low-activity male hemizygotes and low-activity female homozygotes were given a score of 0. *MAOA* variant was treated as a categorical variable in males (0 or 1) and was treated as a continuous variable in females or analyses that included both males and females (0–1).

We used multilevel models (time points nested within participants) to test for effects of *MAOA* genotype, trauma exposure, and resilience on the psychosocial and mental health outcomes over the course of approximately one year. Multilevel models are robust to partially missing data, yielding higher statistical power than linear mixed models and regression analyses, while still taking advantage of a longitudinal design [60, 72]. These features are particularly useful for our data, given that more than half of the participants in this mobile refugee population were lost to follow-up at one year.

All statistical analyses were conducted using R [73]. Multilevel model analysis was conducted using linear mixed effects modeling via the nlme package with measurements at each time point (level one) nested within individuals (level two) [74]. Study site (Irbid, Jarash, Mafraq, Zarqa) and age at T1 were included as fixed effects, and these time-invariant covariates were included in all relevant analyses. Intervention program was treated as a time-varying covariate; all participants were given a score of 0 at T1, while at T2 and T3 those in the control group continued to receive a score of 0 (= has not received the intervention) and those in the treatment group received a score of 1 (= has received the intervention). Measurements of trauma exposure were treated as time-invariant and continuously distributed based on an individual’s score at T1, consistent with previous studies using this sample [57, 75]. Measurements of resilience were treated as time-varying and measurements from all three time points were used. Models that included only Time were used to confirm that sufficient variation existed within the tested outcome to support multilevel modeling. Base models included time and covariates for age, collection site, and intervention. *MAOA*, trauma exposure, and resilience were added to the base models and were used to test for associations between the outcome measure and *MAOA*, trauma exposure, or resilience. Models for perceived psychosocial stress (PSS) were run in the following order (see Table 2):

Model 1. Testing for effects of *MAOA* variant on PSS:

- Time only
- Base (the base model includes time and the covariates: age, site, intervention)
- PSS_*MAOA*_ = Base + *MAOA* + MAOA*Time

Model 2. Testing for effects of *MAOA* variant and Trauma exposure on PSS:

- Time only
- Base (the base model includes time and the covariates: age, site, intervention)
- PSS_*MAOA* + Trauma_ = Base + *MAOA* + MAOA*Time + Trauma + Trauma*Time
- PSS_*MAOA* + Trauma + MAOA*Trauma_ = Base + *MAOA* + MAOA*Time + Trauma + Trauma*Time + MAOA*Trauma + MAOA*Trauma*Time

Model 3. Testing for effects of *MAOA* variant and Resilience on PSS:

- Time only
- Base (the base model includes time and the covariates: age, site, intervention)
- PSS_*MAOA* + Resilience_ = Base + *MAOA* + MAOA*Time + Resilience
- PSS_*MAOA* + Resilience + MAOA*Resilience_ = Base + *MAOA* + MAOA*Time + Resilience + MAOA*Resilience

**Table 2:** Perceived Stress Scale models with male participants.

Perceived Stress Scale (PSS)	Beta	Std. Error	*p* value
**Model 1. Testing for effects of *MAOA* on PSS (n = 221)**			
**Time only**	-0.074	0.016	5.6 x 10^−6^*
**PSS**_***MAOA***_ = Base + *MAOA* + MAOA*Time			
*MAOA*	1.5	0.67	2.4 x 10^−2^
MAOA*Time	0.11	0.033	8.1 x 10^−4^*
**Model 2. Testing for effects of *MAOA* and Trauma exposure on PSS (n = 221)**			
**Time only**	-0.074	0.016	5.6 x 10^−6^*
**PSS**_***MAOA* + Trauma**_ = Base + *MAOA* + MAOA*Time + Trauma + Trauma*Time			
*MAOA*	1.5	0.67	0.025
MAOA*Time	0.11	0.034	7.6 x 10^−4^*
Trauma	0.14	0.11	0.19
Trauma*Time	0.00084	0.0058	0.88
**PSS**_*MAOA* + Trauma + MAOA*Trauma_ = Base + *MAOA* + MAOA*Time + Trauma + Trauma*Time + MAOA*Trauma + MAOA*Trauma*Time			
MAOA*Trauma	0.011	0.21	0.95
MAOA*Trauma*Time	0.0019	0.011	0.86
**Model 3. Testing for effects of *MAOA* and Resilience on PSS (n = 163)**			
**Time only**	-0.061	0.017	3.7 x 10^−4^*
**PSS**_***MAOA***_ = Base + *MAOA* + MAOA*Time			
*MAOA*	2.2	0.78	6.1 x 10^−3^
MAOA*Time	0.11	0.035	1.4 x 10^−3^*
**PSS**_***MAOA* + Resilience**_ = Base + *MAOA* + MAOA*Time + Resilience			
*MAOA*	1.9	0.74	8.6 x 10^−3^
MAOA*Time	0.089	0.034	8.2 x 10^−3^
Resilience	-0.026	0.053	7.9 x 10^−9^*
**PSS**_*MAOA* + Resilience + MAOA*Resilience_ = Base + *MAOA* + MAOA*Time + Resilience + MAOA*Resilience			
MAOA*Resilience	0.057	0.11	0.60

Time only models do not include covariates. Base models include time and covariates for age, study site, and intervention. Significance was tested individually for *MAOA*, Trauma exposure or Resilience with respect to association with the intercept of PSS (reported as *MAOA* or Trauma or Resilience) and for *MAOA* and Trauma exposure for association with the slope of PSS (reported as Trauma*Time or MAOA*Time). *p* values with an asterisk (*) remain significant after correction for multiple testing, i.e. *p* < 1.67 x 10^−3^.

Significance tests for all variables (as reported in Table 2) were conducted using ANOVAs that compared models with and without the variable being tested, i.e. in Model 1 outlined above, the significance of MAOA*Time was tested by comparing models with and without MAOA*Time. Significance after correction for multiple testing was calculated as *p* < 0.05 / (5 psychosocial outcomes with sufficient variation for multilevel modeling x 2 sexes x 3 models) = 1.67 x 10^−3^.

**Fig 1:**
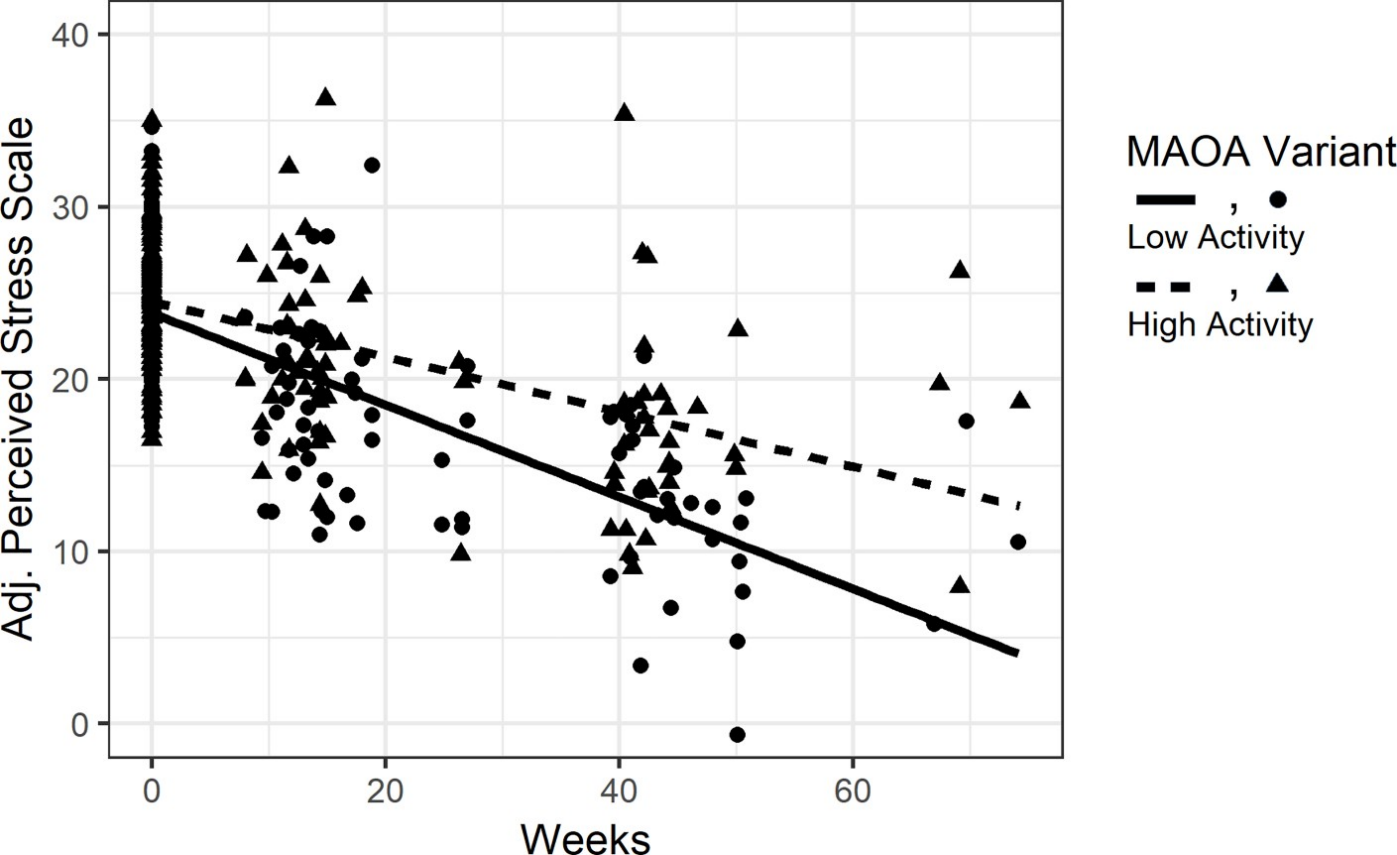
Partial effect plot of perceived psychosocial stress over time by *MAOA* variant in males. *MAOA-L* males had sharper reductions in levels of perceived stress (PSS) over time relative to *MAOA-H* males. Perceived stress symptom scores were adjusted for the effects of all covariates in the model and plotted (Y axis) for each participant at all time points (X axis).

**Fig 2:**
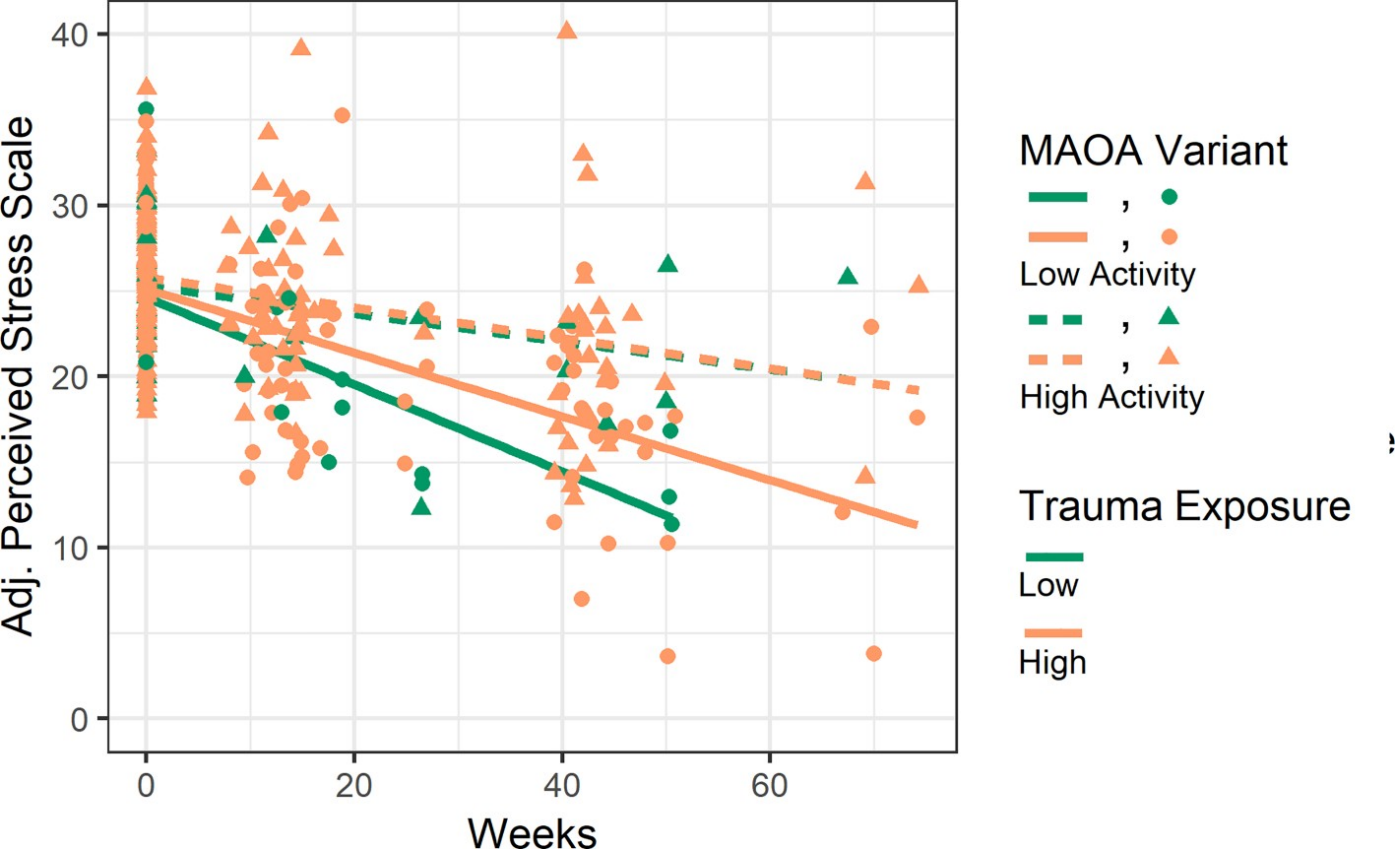
Partial effect plot of perceived psychosocial stress over time by *MAOA* variant and trauma exposure in males. *MAOA-L* males had sharper reductions in levels of perceived stress (PSS) over time relative to *MAOA-H* males. Perceived stress symptom scores were adjusted for the effects of all covariates in the model and plotted (Y axis) for each participant at all time points (X axis). Partial effect plot lines were fitted for the four categories of *MAOA* variant (Low-activity/High-activity) and trauma exposure (Low/High). Trauma exposure was dichotomized around <4 vs ≥4 events in the figure for visualization purposes, but was treated as a continuous measure in multilevel models.

In order to visualize the effects that we report, partial effect plots were constructed using the lme4, remef, and ggplot packages [76–78]. All plots were corrected for the same covariates (age, site, and intervention) that were included in the significance testing. Trauma exposure and resilience scores were treated as continuous variables in the multilevel models and when testing for significance, but were dichotomized when constructing the partial effect plots (Figs 1 and 2) to more clearly illustrate the effects (trauma exposure was dichotomized at <4 vs ≥4 events as this cutoff was previously found to be relevant for this sample [75] and resilience was dichotomized at the median based on averages of all resilience measures for each individual, i.e. median = 51).

## Results

### Sample characteristics

Sample characteristics for the Syrian refugee cohort are shown in Table 1. Genetic data were collected for 221 males and 178 females at T1, with retention of 66% and 39% of participants at T2 and T3, respectively. Exposure to traumatic events was higher for males relative to female participants, but levels of resilience were comparable by gender. No differences in trauma exposure or resilience levels were seen between *MAOA-L* and *MAOA-H* carriers. In terms of outcomes, psychosocial stress (PSS), insecurity (HI), and anxiety and depression (AYMH) symptoms were significantly higher in females.

### Initial analyses by gender

We investigated six measures of psychosocial stress and mental health. All but one outcome (HI) had sufficient variation for multilevel modeling. With the cohort of males and females combined (*n* = 399), we found no significant associations between *MAOA* and the five tested outcomes (PSS, HD, AYMH, SDQ, or CRIES-8). We then divided the sample to examine males and females separately. In females (*n* = 178), no significant associations were found between *MAOA* and the five outcomes. In males (*n* = 221), associations were only significant for symptoms of psychosocial stress as measured on the Perceived Stress Scale (PSS); no associations were found between *MAOA* and either AYMH or SDQ, whilst there was insufficient variation for HD and CRIES-8 to test in multilevel models. Thus, all subsequent analyses focus on PSS in males.

### MAOA

We found that *MAOA* was significantly associated with PSS over time, even after correction for multiple testing (MAOA*Time *p* = 8.1 x 10^−4^; Table 2, Model 1). Plots of *MAOA* and PSS show that *MAOA* variants exhibited different changes in PSS over time (Fig 1). Specifically, *MAOA-L* males had sharper reductions in PSS levels over time as compared to high *MAOA-H* males.

### *MAOA* and trauma exposure

We next tested to see if the association identified above between *MAOA* and PSS was influenced by trauma exposure. Although previous studies have found interactive effects between *MAOA* and trauma, we found no interactive effect of *MAOA* and trauma exposure on PSS (MAOA*Trauma *p* = 0.95 and MAOA*Trauma*Time *p* = 0.86; Table 2, Model 2). As expected, *MAOA* was still directly associated with PSS over time, even with trauma exposure included in the model (MAOA*Time *p* = 7.6 x 10^−4^; Table 2, Model 2). Plots of *MAOA* and trauma exposure show that *MAOA-L* males had the sharpest reduction in PSS (Fig 2). Furthermore, there appeared to be an additive effect wherein *MAOA-L* males with low trauma exposure had the sharpest reductions in PSS over time, as compared to all *MAOA-H* males and *MAOA-L* males with high trauma exposure. In contrast to the difference in PSS shown by *MAOA-L* males with respect to trauma exposure, *MAOA-H* males with high and low trauma exposure showed the same PSS trajectory.

### *MAOA* and resilience

**Fig 3:**
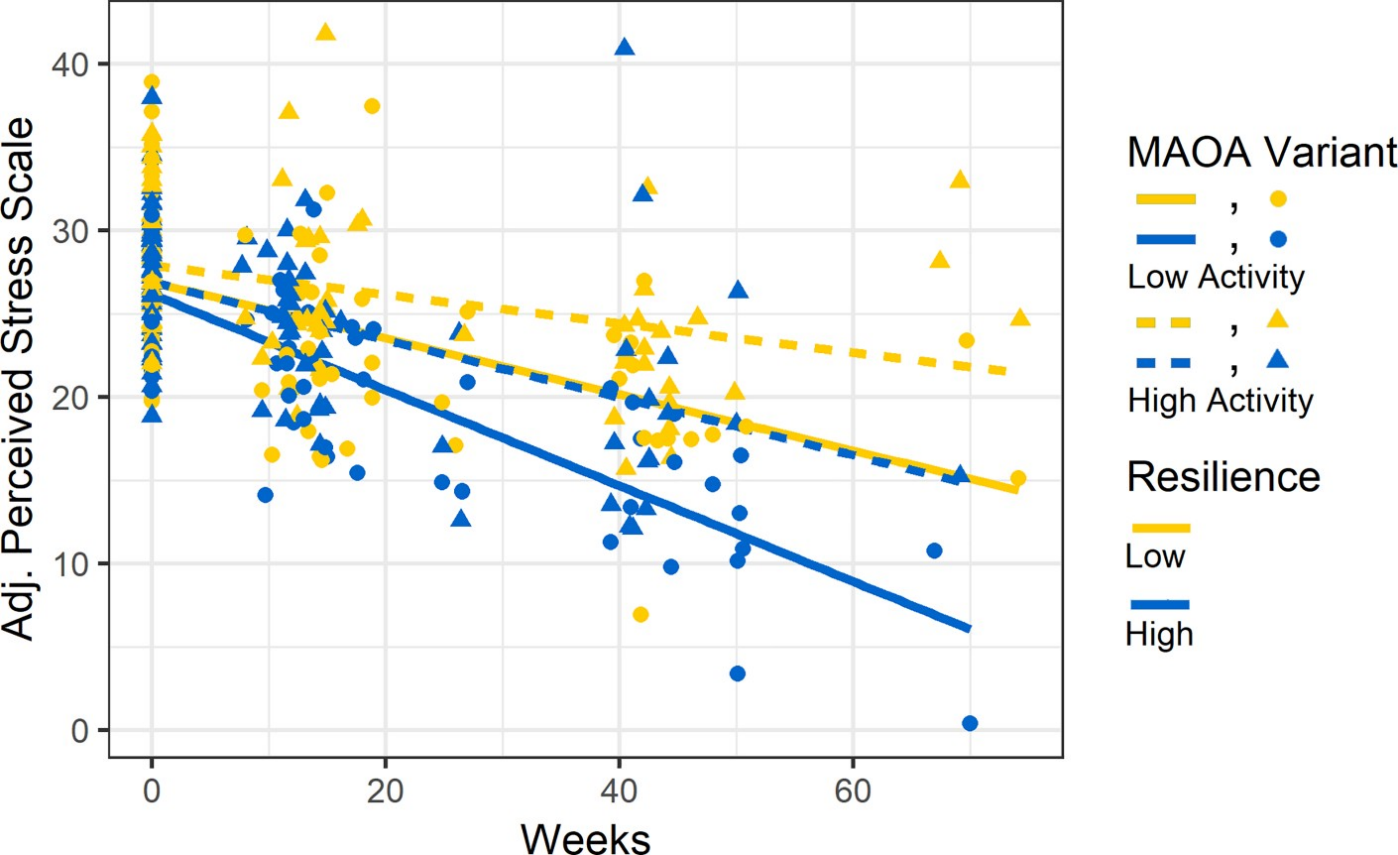
Partial effect plot of perceived psychosocial stress over time by *MAOA* variant and resilience levels in males. *MAOA-L* males with high resilience had the sharpest reduction in levels of perceived stress (PSS) over time. Perceived stress symptom scores were adjusted for the effects of all covariates in the model and plotted (Y axis) for each participant at all time points (X axis). Partial effect plot lines were fitted for the four categories of *MAOA* variant (Low-activity/High-activity) and Resilience (Low/High). Resilience was dichotomized around the median (median = 51) in the figure purely for visualization purposes but was treated as a continuous measure in multilevel models.

We next tested to see if the association between *MAOA* and PSS was influenced by participants’ levels of resilience. We first confirmed that the direct effect of *MAOA* on PSS over time reported above was also present in the smaller sample with resilience data (MAOA*Time *p* = 1.4 x 10^−3^; Table 2, Model 3). When levels of resilience were added to the model, we found that *MAOA* was still associated with PSS, but no longer survived our conservative correction for multiple testing (MAOA*Time *p* = 8.2 x 10^−3^; Table 2, Model 3). We also found that resilience was strongly associated with PSS (Resilience *p* = 7.9 x 10^−9^; Table 2, Model 3). We found no significant interactive effects between *MAOA* variants and resilience on PSS (*MAOA* x Resilience *p* = 0.28; Table 2, Model 3). Plots show that higher levels of resilience were associated with lower PSS at baseline and with a sharper reduction in PSS over time (Fig 3). Furthermore, there appeared to be an additive effect with resilience wherein *MAOA-L* males with high resilience had the sharpest reductions in PSS over time compared to all *MAOA-H* males and *MAOA-L* males with low resilience (Fig 3).

## Discussion

This is the first study to test the effect of early life trauma exposure, *MAOA* genetic variants, and a culturally-specific measure of resilience on psychosocial outcomes in a population of Syrian refugee youth over time. Specifically, we tested for direct and interactive effects of *MAOA* variants, trauma exposure, and resilience levels on psychosocial outcomes at baseline and over approximately one year. We tested a range of psychosocial and mental health outcomes in order to better understand the range of behaviors that may be influenced by trauma, *MAOA*, and resilience.

We found a significant association between *MAOA* and perceived psychosocial stress (PSS) over time, in males only, which remained significant after strict Bonferroni correction for multiple testing (MAOA*Time *p* = 8.1 x 10^−4^; Table 2, Model 1). We found direct effects of *MAOA* on PSS, but no interactive effects between *MAOA* and trauma. We also found no associations between *MAOA* and the tested outcomes in the overall cohort, or in females only. These results suggest that *MAOA* plays a role in influencing levels of psychosocial stress in the males in our study population independent of their trauma exposure. Furthermore, *MAOA* was only significantly associated with changes in PSS over time (MAOA*Time *p* = 8.1 x 10^−4^ vs *MAOA p* = 2.4 x 10^−2^; Table 2). Specifically, *MAOA-L* males showed sharper reductions in PSS scores over time (Fig 1). The fact that the association of *MAOA* and PSS only emerges over time highlights the importance of conducting longitudinal research in order to understand the long-term effect of *MAOA* on psychosocial stress and possibly other measures of mental health.

We report no significant association between trauma and PSS in the males in our study population. However, we have found a significant association between trauma and PSS in the larger dataset of all Syrian refugees including those with no biological samples (n = 446) (unpublished data), so it may be that our smaller sample size of males only (n = 221) is not sufficient to detect an association between trauma and PSS. Interestingly, if we treat trauma as time-varying in the multilevel model, we do detect an association between trauma and PSS in males, although the *p* value (*p* = 0.014) is not significant after correction for multiple testing. This result suggests there is a trend of trauma exposure impacting PSS although the effect is not straightforward.

We also found that a culturally-relevant measure of resilience (CYRM-12) was strongly associated with psychosocial stress (*p* = 7.9 x 10^−9^; Table 2, Model 3). When resilience was added to the model, *MAOA* was no longer associated with PSS (after correction for multiple testing). Furthermore, we saw an additive effect with both trauma exposure and resilience levels wherein *MAOA-L* males with either low trauma exposure or high resilience showed the sharpest reductions in psychosocial stress (Figs 2 and 3).

Very few studies of complex phenotypes, such as response to trauma, include both genetic and psychosocial factors even though both genetic and environmental factors are involved in all complex phenotypes. By including both types of factors, we identified additive effects between *MAOA* and trauma exposure and between *MAOA* and resilience on psychosocial stress. Additive effects act independently, and not as gene x environment interaction effects wherein the environmental factor exerts different effects on an individual based on his or her genotype. Furthermore, by comparing the effects of *MAOA* and resilience, we conclude that *MAOA* variation impacts levels of psychosocial stress in males, but that resilience appears to be a more influential factor.

Our study is the second to examine the effect of *MAOA* and resilience on mental health outcomes. We find an additive effect wherein *MAOA-L* males with high resilience showed the sharpest reductions in perceived psychosocial stress (Fig 3). The only other study to investigate the impact of protective factors and *MAOA* variants on mental health found a similar result with *MAOA-L* carriers. Specifically, Kinnally et al. [40] found that high perceived parental care mitigated the effect of a childhood stressor on impulsivity scores in *MAOA-L* females (males were not studied); level of perceived parental care had no effect on impulsivity in homozygous *MAOA-H* females.

Our results are consistent with Pluess and Belsky’s model of environmental sensitivity that posits carriers of sensitivity variants are more sensitive to both positive and negative environmental exposures [52, 53, 79]. Pluess [51] proposed a theoretical framework in which environmental sensitivity is a function of specific genetic variants, and genetic factors predict individual differences in environmental sensitivity to both positive and adverse conditions. Belsky and Pluess and colleagues [80, 81] specifically proposed that due to genetic differences, some children would benefit more from intervention efforts and that evaluations of intervention efficacy should account for such genetic variation. In the case of *MAOA*, our results suggest that *MAOA-L* variants may function as sensitivity variants and carriers may be more responsive to the effect of both negative (trauma exposure) and positive (levels of resilience) factors. Similarly, other studies have found that the *MAOA-L* variant is most sensitive to a positive factor (high perceived parental care; [40]) and to negative factors (aggressive and antisocial behavior in the presence of childhood trauma [17, 25, 82, 83]). Furthermore, in a brain imaging study, Buckholtz et al. [28] proposed that *MAOA-L* individuals may be more susceptible to environmental factors than *MAOA-H* individuals due to their amygdala dysregulation and compensatory response between the amygdala and the ventromedial prefrontal cortex. It is important to note that Pluess [51] posited that sensitivity variants act through a gene x environment mechanism with no proposed difference between genetic variants in the absence of adverse or positive conditions. We found a direct effect of *MAOA* on PSS, rather than gene x environment effects with trauma exposure or resilience, which may suggest that sensitivity variants can also act through an additive mechanism.

As noted above, we only identified associations of *MAOA* and resilience with PSS in males. This result is consistent with other studies that have found the effect of *MAOA* on measures of psychosocial health and behavior to be weak or non-existent in females [28, 39, 83, 84]. Since *MAOA* occurs on the X chromosome, males and females differ in copy number of *MAOA* and random X chromosome inactivation in females means that females likely have variable MAOA levels throughout the body relative to males. Studies of brain morphology and brain activity have found different effects of childhood trauma on male and female brains [23, 27] so *MAOA* may have different biological effects in males and females.

There are three main limitations to note in our study. First, we focused on a single gene. *MAOA* is a well-supported candidate gene to investigate the impact of childhood trauma, but additional genes should be studied in order to fully understand the impact of childhood trauma and resilience on adult behaviors, particularly in refugee populations with exposure to high levels of trauma. Second, our focus on Syrian refugees, who have relatively high levels of trauma exposure, may have restricted the level of population variation in some of the psychosocial health outcomes. Third, our sample size is small, particularly since the association we identified was only found in males, which reduced our sample size. A recent meta-analysis found no support for 18 previously identified candidate genes for depression, suggesting that previous studies had been underpowered [85]. Research on high trauma populations can help us understand the impact of trauma on mental health, but collection of a large number of longitudinal samples from a highly mobile refugee population will always be challenging [86]. The fact that, even after correction for multiple testing, we detected significant associations with psychosocial stress in our study population is encouraging. Nevertheless, work with a larger, more heterogeneous sample is needed to more fully evaluate the effects of *MAOA*, trauma exposure, and resilience on psychosocial stress and mental health measures. In order to fully understand the impact of trauma on mental health, more work with vulnerable populations is necessary, particularly in non-Western contexts with high levels of trauma exposure.

## Conclusions

We report significant effects of *MAOA* genetic variants and resilience levels on changes in perceived psychosocial stress over time, in a population of Syrian refugee male youth. We also find an additive effect wherein males with low-activity *MAOA* variants who reported low trauma exposure or high resilience had the sharpest reductions in perceived psychosocial stress over time. Our results highlight the potential scientific value of careful and ethical collection of data and biological samples in populations with high levels of trauma exposure. In contexts of war, violence, and forced migration, addressing the root causes of human suffering and the health consequences of toxic stress and trauma exposure is of vital importance. It is also prudent to reach for an in-depth understanding of the interplay between genetic and psychosocial factors that are able to influence the expression of interpersonal and social behaviors over the life course.

## Supporting information

S1 Table Data on trauma exposure, resilience level, psychosocial stress and mental health measures, and MAOA variants for all study participants. See S2 Table for codebook for variable names presented here.
(CSV)

S2 Table Codebook for variable names presented in S1 Table. (XLSX)
